# Examining the Connection between Religion and Attitude toward Socio-Economic Human Rights and Attitude toward Euthanasia and Abortion among Romanian Orthodox Adolescents: Contrasting the Effects of Intrinsic and Extrinsic Religiosity

**DOI:** 10.3390/ijerph182010837

**Published:** 2021-10-15

**Authors:** Silviu E. Rogobete, Leslie J. Francis, Ursula McKenna

**Affiliations:** 1 Department of Political Science, West University of Timisoara, 300223 Timisoara, Romania; 2 Centre for Educational Development, Appraisal and Research, University of Warwick, Coventry CV4 7AL, UK; Leslie.Francis@warwick.ac.uk; 3 World Religions and Education Research Unit, Bishop Grosseteste University, Lincoln LN1 3DY, UK; ursula.mckenna@bishopg.ac.uk

**Keywords:** religious orientation, church attendance, prayer, human rights, euthanasia, abortion

## Abstract

This study examines the complex connection linking religion, social attitudes, and human rights in Romania, drawing on the classic distinction between extrinsic religiosity (as reflected in church attendance) and intrinsic religiosity (as reflected in personal prayer). The hypothesis that these forms of religiosity may function differently in relation to different areas of social attitudes is tested among Romanian Orthodox adolescents (N = 400), drawing on validated measures developed by the International Empirical Research Program Religion and Human Rights 2.0 to assess attitude toward socio-economic human rights and attitude toward euthanasia and abortion. In respect of attitude toward euthanasia and abortion, church attendance and personal prayer work in the same direction and with cumulative effect. Lowest acceptance of euthanasia and abortion is found among young people who attend church and pray. In respect of attitude toward socio-economic human rights, church attendance and personal prayer work in opposite directions. Frequent church attendance (extrinsic religiosity) is associated with lower endorsement of socio-economic human rights. Frequent prayer (intrinsic religiosity) is associated with higher endorsement of socio-economic human rights.

## 1. Introduction

### 1.1. Religion, Bioethics and Social Values in Romania

Despite a growing volume of academic literature on the return of religion in contemporary society [1], empirical research assessing the importance of the return of religion for adolescents is still in its early stages. This is also true in relation to the study of the influence of religion on adolescents’ understanding of and support for various forms of human rights and social values. The present study proposes an empirical assessment of the impact of some aspects of religiosity on issues related to matters of bioethics (abortion and euthanasia), and on some specific socio-economic rights. The study was conducted in Romania, a country with some particularities providing for a highly relevant case for at least three main reasons.

First of all, Romania is the most religious among 34 European countries, according to current empirical research [2]. Secondly, Romania is a country with a slow record of democratisation processes and the implicit human rights culture [3,4]. Thirdly, issues related to bioethics are under-researched and under-developed, compared to other EU countries [5]. In order to provide the wider context for the current study, we will further elaborate on each of these three points in turn, looking also at some of the dynamics between them.

In terms of religion, Romania is a majority Eastern Orthodox Christian country. As the second largest Christian denomination worldwide after the Roman Catholic Church, Eastern Orthodoxy is a traditional denomination rooted in the Early Church, today being a majority denomination in Greece and almost all the Slavonic countries. One major distinguishing characteristic is its form of governance, the so-called auto-cephaly, meaning that each national Orthodox church has its own “head”, the Patriarch. This leads, among other things, to very strong ties between religious and national identity [6,7]. In Romania, roughly constant figures for the last three decades show that about 82% of the population belong to the Romanian Orthodox Church, and over 98% belong to a Christian religious denomination. The percentage of assumed atheists remains extremely low, with 0.05% in 1992, 0.04% in 2002, and 0.11% in 2011 according to the three national official censuses run since 1989, the year marking the end of fifty years of totalitarian atheistic indoctrination [8]. Although assessments of the religious landscape of Romania have been made from various angles [6,9,10,11] of relevance for this particular study are data related to the distinction between extrinsic and intrinsic religiosity [12].

Evans and Baronavski [2] found Romania as the top most religious among 34 other European countries based on an index created by the combination of four individual measures of religious observance: self-assessment of religion’s importance in one’s life, religious attendance, frequency of prayer, and belief in God. According to this index, 55% of adults in Romania are “highly religious”. For 50% religion is very important in their lives, and in terms of church attendance, 50% of Romanians also say they attend worship at least monthly. Such findings of high levels of religiosity are highly consistent with national surveys [13]. However, this must be seen and assessed in the light of other figures, potentially pointing to the use of religion as means for external ends, rather than just for its own sake. For instance, the same international study shows that 76% of the respondents consider that to be a good Romanian and share in Romanian national identity, one has to be an “Orthodox Christian”. This places Romania again at the top of the international chart, followed by Bulgaria, Armenia, Georgia, and Serbia, all similarly majority Orthodox countries in agreement with the importance of their Christian faith for sharing in one’s national identity [2]. This is in strong contrast with Western European countries, for most of which being a Christian is not a very or not at all important part of the national identity [14]. Moreover, this study also shows another aspect pointing to the extrinsic use of religiosity in predominantly Orthodox countries. Self-perceived national cultural superiority seems to be higher in countries where Orthodoxy is the majority religious denomination. All three top respondents in agreement with the statement: “Our people are not perfect, but our culture is superior to others” belong to Orthodox nations: Greece (89%), Bulgaria (69%), and Romania (66%). No Western European country assessed agreed with this statement in proportions above 50% [14].

In terms of religiosity as a more personal, intrinsic endeavour, 44% of Romanians say they pray daily and 64% claim to believe in God with absolute certainty. A more detailed national survey adds that in Romania 96% believe in God, 60% say they have their personal belongings blessed by a priest, 60% believe that “heavens” exist, and 54% believe in life after death [13].

In terms of social values and respect for the rights of others, Romanians, for instance, are less willing than Western Europeans to say they accept Muslims or Jews as members of their family or as neighbours: only 29% Romanians (31% Greeks, 32% Bulgarians, and 32% Poles) would be willing to accept Muslims as relatives. At the top of this chart is The Netherlands, with 88% accepting. Similar contrasts apply when endorsing same-sex marriage, Romania coming fourth in terms of the lowest percentage, with 26% in favour, while Bulgaria has 18%, Latvia 16%, and Lithuania 12%. Sweden and Denmark are at the top, with 88% and 86% respectively in favour of it [2]. Such low levels of support for alterity, often also found in hatred discourse, is well reflected in regular national polls in Romania [15].

In more general terms, human rights and particularly individual rights were considered as imperialistic ideology of the West during the cold war [16], thus being a relatively new concept for Romanian society. With the required democratisation processes in place, however, Romania became rather quickly a member of the Council of Europe (1993) and a signatory of the European Convention of Human Rights (EConvHR), thus placing all issues related to human rights under the jurisdiction of the European Court of Human Rights (ECHR). Moreover, becoming a member of the European Union (2007), Romania is also under the jurisdiction of the European Court of Justice. However, currently Romania and Bulgaria are the only countries still formally monitored on judicial matters by the EU Commission, 14 years after they formally joined the EU [17].

Hence, the jurisprudence of the ECHR, various International Non-Governmental Organisations, and the US State Department’s annual country reports on human rights show progress, yet some significant issues still remain [18]. They are related to the restitutions of property seized by the totalitarian regime in the 1950s, and minority rights (sexual, religious, and ethnic—particularly Roma communities). Moreover, among all the EU states, Romania is currently one of the countries with the highest numbers involved in human trafficking [19].

### 1.2. Bioethics, with a Special Emphasis on Abortion and Euthanasia

Bioethics is a relatively new domain in Romanian academia and legal practice. Loue [5] shows that at the time of her study, no major medical university in Romania had a full compulsory course on Bioethics. By 2018 there were a few medical schools offering courses on bioethics, with an emphasis on legal medical practice rather than moral and philosophical issues related to life and death [20]. As such, informed debates on matters like assisted reproduction technology, abortion, or euthanasia are either neglected or left to religious communities and secular activists [21]. From a political perspective, there were some legal initiatives to define and regulate the complex field of bioethics that have reached Parliament, but were eventually abandoned [22]. The only legal framework in place is the National Commission on the Bioethics of Medicines and Medical Devices (OM 1446/2009). However, as the title suggests, it is restricted to the use of medicines and medical devices, leaving unanswered most of the major ethical questions related to the complex field of bioethics. Some of these matters are only dealt with at the level of every day medical practice, and at institutional level by the medical professional association, the Romanian College of Physicians. It is done mainly under the guiding principles of the Convention on Human Rights in the Field of Bioethics of the Council of Europe, to which Romania is a part and according to their own Code of Medical Deontology. Nevertheless, the President of the College has pointed out in an interview that Romania is the only EU country without a national committee on bioethics with formal legal and political powers [23].

The radical laws against abortion in place up until 1989 made Romania move in the opposite direction. Within one day from the fall of Ceausescu’s regime, the first Decree issued by the new government (Law nr. 1/26 December 1989) included the law permitting abortions (Article 8). The immediate consequence put Romania at the top of the list, with the highest number of abortions performed at European (and in the 1990s even global) level. Even if currently somewhat lower, despite such high levels of religiosity as seen above, abortion is still widely practised, being used as one of the main means of contraception when compared to other EU countries [24]. Currently, abortions are legally regulated by the Penal Code (Legea 286/2009), under the heading “Aggressions against the human foetus” (Articles 201–202). Abortion is thus legal up to the fourteenth week of pregnancy, and at later stages only based on professionally determined medical reasons. The mother, nevertheless, can perform an abortion herself at any stage of her pregnancy, without being prosecuted (Article 201.7), and it is not considered a crime for the mother if she harms the foetus during pregnancy (Article 202.7). Medical staff are nevertheless free to refuse to perform abortions, something often found in current Romanian public and private practices.

Euthanasia is legally forbidden in Romania under any circumstances, being considered murder. The New Penal Code (Legea no. 286/2009) deals with this matter in Article 190, under the heading “Killing at the victim’s request” and stipulates that: “Killing at the explicit, serious, conscious and repeated requests of the victim suffering of an incurable illness or having a grave medically attested infirmity, causing permanent and unbearable suffering, is punished with prison from 1 to 5 years”. The topic, with arguments for and against euthanasia, is nevertheless at the centre of various academic and religious debates in Romania [25].

### 1.3. Connecting Religion with Human Rights, Abortion and Euthanasia

The high level of religiosity is perhaps best reflected in the constant presence and the strong influence of the Romanian Orthodox Church in all major aspects of social and often political life, including the politics related to bioethics [26,27]. The position held on abortion and euthanasia is very firm against both, in almost any form and under any circumstances. Iloaie [21] provides an accurate survey of the official position of the Romanian Orthodox Church on bioethics. In 2001 a National Consultative Commission of the Romanian Orthodox Church on Bioethics was formed. The Synod of the Church has since issued three separate documents based on the proposals from the commission, dealing in turn with abortion, euthanasia, and organ donation and transplant. As it is expected, the documents contain theological, moral, and religious traditional arguments in favour of life. Thus, euthanasia is completely forbidden, while the position on abortion and organ donation are somewhat more nuanced. In regard to abortion, the rights of the unborn prevail, abortion performed in any form being considered an interference with the life of the unborn, which is ultimately murder. Under circumstances where the life of the mother is put in danger due to pregnancy, abortion is nevertheless permitted by the Church. A strong emphasis is placed on support offered by the Church and the state and on education and information related to the Church’s position on both abortion and euthanasia.

Various aspects of the position of the majority Romanian Orthodox Church on issues related to human rights were already assessed in some detail elsewhere [9,28,29]. The Synod of the Romanian Orthodox Church has not issued any formal position on the topic of human rights, as for instance the Russian Orthodox Synod did in 2008 [30]. Formally, at least at the official discursive/theological level, human rights are not overtly rejected. To the contrary, the current head of the Church, Patriarch Daniel, endorsed and praised the notions of human dignity encompassed in the Universal Declaration of Human Rights marking 60 years since its adoption [31]. Also, some theologians present friendly perspectives on various aspects of human rights [32].

There is, however, a highly dominant trend that rejects the discourse of human rights. The majority of the priesthood, and particularly the powerfully influential monastics, see and present human rights as a Western European ideology, rooted in the secular Enlightenment and therefore an alien teaching, if not altogether an enemy of the doctrines of the Eastern Orthodox Church and its traditions [33]. This is particularly the case when human rights discourse is used to support the rights of various minority groups and individual rights, based on issues like sexual orientation or feminist ideology. There are many internet sites where traditional orthodox teaching is critical of human rights. Their teachings become highly visible through periodical public manifestations, powerful petitions, and lobbying against claims for such rights. The most recent was the national referendum for the redefinition of the notion of family in Romanian law. There are, nevertheless, a few theologians and perhaps a growing number of faithful Orthodox lay intellectuals who see the question of human rights as an affirmation of human dignity and therefore somehow in consonance with the Christian faith. Yet, their influence within the church is still rather limited.

In conclusion, Romania is a highly religious country, with a powerful and highly influential majority Orthodox Church. At the same time, abortion is widely practiced, the culture of human rights is still weak, respect for alterity and the rights of various minorities is lacking, and corruption levels are still high. Such a combination certainly raises a series of questions related to the nature of the religiosity claimed by its followers. Is such religiosity a mere external ideological replacement of a failed dominant political ideology, with no personal appropriation of its values and the subsequent translation into social values and norms? How do Romanian adolescents relate to religion, and what is its role in the promotion of and support for a culture of human rights and social values? To such questions we will attempt to offer some possible answers based on this current research. Before moving into the concrete details of the research itself, we reflect on the theoretical framework used.

### 1.4. Religious Orientation Theory

The connection between social values and religion was cast in a new light during the 1950s and 1960s by Gordon Allport. Allport recognised that the contradictory effects of religion on social values could be clarified by distinguishing between two fundamentally different religious orientations and motivations. Allport styled these two orientations as intrinsic religiosity and extrinsic religiosity. According to Allport, the distinction between extrinsic and intrinsic religiosity distinguished between “churchgoers whose communal type of membership supports and serves other, non-religious ends, from those for whom religion is an end in itself—a final, not instrumental good” [12] (p. 455).

Developing and extending Allport’s model of religious orientation, Batson [34] and Batson and Ventis [35] proposed a third orientation alongside the intrinsic and extrinsic orientations, which they styled the quest orientation. The quest orientation acknowledged a form of religiosity that embraces characteristics of complexity, doubt, tentativeness, and honesty in facing existential questions.

Several different instruments have been developed to measure the three components of religious orientation theory, including the scales of intrinsic orientation and extrinsic orientation proposed by Allport and Ross [36], the original measure of quest orientation proposed by Batson and Ventis [35], the revised measure of quest proposed by Batson and Schoenrade [37,38], and the New Indices of Religious Orientation (NIRO) proposed by Francis [39].

While the introduction of the notion of quest religiosity amplified religious orientation theory, it did not serve well in clarifying the relationship between religion and social values. The distinction between intrinsic religiosity and extrinsic religiosity has remained fundamental in this regard. While the development of psychometrically sound measures of intrinsic religiosity and extrinsic religiosity have clearly advanced empirical research in the field, brief behavioural measures have also been found to serve as reliable and valid operationalisations of the distinction between intrinsic religiosity and extrinsic religiosity. In this regard public worship attendance serves as a good proxy measure for extrinsic religiosity and personal prayer serves as a good proxy measure for intrinsic religiosity.

### 1.5. Research Question

Against this background, the aim of the present study is to test the thesis that intrinsic religiosity (as measured by frequency of personal prayer) and extrinsic religiosity (as measured by frequency of worship attendance) are related in opposite directions in respect of attitude toward socio-economic human rights among Romanian Orthodox adolescents. The hypotheses are that high levels of church attendance are associated with lower endorsement of socio-economic human rights, and that high levels of personal prayer are associated with higher endorsement of socio-economic human rights. The specificity of these hypotheses to socio-economic human rights is tested against a second variable, attitude toward euthanasia and abortion, where it is hypothesised that intrinsic religiosity and extrinsic religiosity work in the same direction. The hypotheses are that high levels of church attendance are associated with lower endorsement of euthanasia and abortion, and that high levels of personal prayer are also associated with lower endorsement of euthanasia and abortion. Moreover, it is further hypothesised that these effects are cumulative, so that low levels of endorsement of euthanasia and abortion are associated with high frequency of church attendance coupled with high frequency of personal prayer.

## 2. Materials and Methods

### 2.1. Research Context: Religion and Human Rights 2.0 Empirical Project

The current paper is part of the International Empirical Research Program Religion and Human Rights 2.0 [40]. The program provides an apt environment in which to test the effect of different religious measures on attitudes toward socio-economic human rights and on attitudes toward euthanasia and abortion, since this program included theoretical discussion of and measures to operationalise both constructs. The program focused on adolescence as an age-group formative for the international future.

Thus, in terms of attitudes toward socio-economic human rights, the International Empirical Research Program Religion and Human Rights 2.0 identified seven main areas and proposed two items to operationalise each area: the state’s obligation regarding the right to work, the state’s obligation regarding the right to social security, the state’s obligation regarding living wages, the state’s obligation regarding rest and leisure, the state’s obligation regarding the rights of children, the state’s obligation regarding the protection of women from discrimination, and the state’s obligation regarding the protection of homosexuals from discrimination [40]. Testing the scaling properties of these 14 items on data provided by a sample of 987 students between the ages of 14 and 18 years in England and Wales, Francis, McKenna, and Sahin [41] reported a high alpha coefficient of .89. At the same time they found that the scaling properties of the instrument could be improved by omitting the two items relating to homosexuals. Francis, McKenna, and Sahin [41] employed this instrument to explore the association between attitudes toward socio-economic human rights and three dimensions of religion (religious practice, religiosity, and self-assigned religious affiliation), after taking into account personal factors (sex and age), and psychological factors (extraversion, neuroticism, and psychoticism).

Working within the same framework of the International Empirical Research Program Religion and Human Rights 2.0, Rogobete and Vitelar [11] tested the scaling properties of a somewhat different set of 12 items related to socio-economic rights on data provided by a sample of 681 Romanian adolescents, reporting a high alpha coefficient of .88. Using this instrument, Rogobete and Vitelar [11] enquired if religiosity has any influence on supporting socio-economic rights, as well as exploring the respondents’ perception of the role of the state in securing such rights. They were also interested in understanding the nature of the relationship between religiosity (including the perception of the functions of religion), family background, and the socio-psychological and political traits of the respondents, and their support for socio-economic rights.

In terms of attitude toward euthanasia and abortion, the International Empirical Research Program Religion and Human Rights 2.0 identified seven items relating to abortion. One item concerned total prohibition of abortion and six items concerned grounds on which abortion may be permitted: in the case of rape, in the case of incest, when there is a strong chance of serious defects to the baby, when the woman’s own health is seriously endangered by the pregnancy, when the woman cannot afford more children economically, and when the woman cannot afford more children psychologically. The program also identified three items relating to euthanasia. One item concerned total prohibition of euthanasia and two items concerned grounds on which euthanasia may be permitted: in the case of unbearable and irreversible suffering, and in the case of palliative care being exhausted. Testing the scaling properties of these ten items on data provided by 966 students between the ages of 14 and 18 years in England and Wales, Francis, McKenna, and Sahin [42] reported a high alpha coefficient of .87. Francis, McKenna, and Sahin [41] employed this instrument to explore the association between attitudes toward euthanasia and abortion and both religious practices (personal prayer and worship attendance) and self-assigned religious affiliation (Christian Protestant, Christian Catholic, Muslim, and religiously unaffiliated), after taking into account personal factors (age and sex), and psychological factors (extraversion, neuroticism, and psychoticism).

Working within the same framework of the International Empirical Research Program Religion and Human Rights 2.0 Breskaya, Botvar, Sjöborg and Rogobete [43] employed the same instrument in an international comparative study assessing, among other aspects related to religion and human rights, the ways in which religious affiliation plays a role in generating various attitudes towards abortion amongst adolescents from four countries: Sweden, Norway, Belarus, and Romania.

### 2.2. Procedure and Participants

Within the context of the International Empirical Research Program Religion and Human Rights 2.0, students attending high schools from the capital cities of the Development Regions of Romania (Timisoara, Cluj-Napoca, Iasi, Constanta, Sibiu, Ploiesti, and Craiova) and from Romania’s capital (Bucharest) were invited during the period September to December 2015 to complete an online survey. The decision to be completed online rather than pen-and-pencil was to take advantage of the study population and the study setting, as well as significantly reducing the costs involved [44]. Thus, the questionnaire, translated into Romanian (using forward and back translation, sector-specific knowledge, and mother tongue translators), was completed during regular participation in Information Technology classes, under the supervision of the class instructors. All students were given the option not to participate, thus participation in the research was voluntary, totally anonymous, and based on informed consent. A total of 681 students across 12 schools completed the survey. The analyses reported in this paper were conducted on the data provided by the 400 students who self-identified as members of the Romanian Orthodox Church, and who fully completed all the measures employed in the analysis. This group comprised 217 females and 183 males, 41 students aged 15 years, 108 aged 16 years, 141 aged 17 years, and 11 aged 18 years.

### 2.3. Ethics Statement

The research conducted in the present study is in full compliance with all standards of good scientific practice and ethical codes expressed in the documents of the German Science Foundation. Letters for Research Ethical Codes Compliance regarding the International Religion and Human Rights Research program were issued both by the Julius-Maximillian University of Wurzburg and by the West University of Timisoara (code 7/2015/CEDU-UVT) and are available upon request. The ethical guidelines maintain that the school operating *in loco parentis* is able to authorise participation of students within the survey conducted with educational intentions and with full confidentiality and anonymity afforded.

### 2.4. Measures

*Attitude toward socio-economic human rights* was assessed by a 12-item scale, drawn from the International Empirical Research Program Religion and Human Rights 2.0, designed to operationalise six specific issues: the state’s obligation regarding the right to work, the state’s obligation regarding the right to social security, the state’s obligation regarding living wages, the state’s obligation regarding rest and leisure, the state’s obligation regarding the rights of children, and the state’s obligation regarding protection of women from discrimination (see [41]). Each of these six areas was operationalised by two items. Each item was rated on a five-point Likert scale: disagree strongly (1), disagree (2), not certain (3), agree (4), agree strongly (5).

*Attitude toward euthanasia and abortion* was assessed by a 10-item scale, drawn from the International Empirical Research Program Religion and Human Rights 2.0, comprising three items about euthanasia and seven items about abortion (see [42]). Each item was rated on a five-point Likert scale: disagree strongly (1), disagree (2), not certain (3), agree (4), and agree strongly (5).

*Personal factors* were assessed by two variables: sex, male (1) and female (2); and age, 15 years (1), 16 years (2), 17 years (3), and 18 years (4).

*Frequency of worship attendance* was assessed by the question “How often do you take part in religious services at a church or mosque or another place?” rated on a six-point scale: never (1), hardly ever (2), a few times a year (3), 1–3 times a month (4), once a week (5), and more than once a week (6).

*Frequency of personal prayer* was assessed by the question “How often do you pray?” rated on an eight-point scale: never (1), hardly ever (2), a few times a year (3), 1–3 times a month (4), once a week (5), more than once a week (6), once a day (7), and several times a day (8).

*Religious saliency* was assessed by the question “How often do you think about religious issues?”, rated on a five-point scale: never (1), rarely (2), occasionally (3), often (4), and very often (5).

*Religious openness* was assessed by the question, “How often do you reconsider certain aspects of your religious views?”, rated on a five-point scale: never (1), rarely (2), occasionally (3), often (4), and very often (5).

*Religious belief* was assessed by the question, “I believe that God is the foundation of everything that exists”, rated on a five-point scale: totally disagree (1), disagree (2), not sure (3), agree (4), and fully agree (5).

### 2.5. Analysis

The data were analysed by SPSS (IBM SPSS Statistics for Windows Version 24, IBM Corp, Armonk, NY, USA) using the frequency, reliability, correlation, and regression routines.

## 3. Results

The first step in data analysis examined the scale properties among Romanian Orthodox adolescents of the two scales drawn from the International Empirical Research Program Religion and Human Rights 2.0. The data presented in Table 1 examines the 12-item Scale of Attitude towards Socio-economic Human Rights (SASHR) in terms of the correlations between the individual items and the sum of the other eleven items, and in terms of the item endorsements expressed as the sum of the agree and agree strongly responses. The correlations ranging from .24 to .76 demonstrate that the majority of the items worked well together to produce a homogeneous scale. The one weak item was “Employment without paid holiday leave should be forbidden” (*r* = .24). The alpha coefficient [45] demonstrated a high level of internal consistency reliability (α = .90, Mean = 48.6, SD = 7.0).

The data presented in Table 2 examines the 10-item Scale of Attitude toward Euthanasia and Abortion (SAEA) in terms of the correlations between the individual items and the sum of the other nine items, and in terms of the item endorsement expressed as the sum of the agree and agree strongly responses. The correlations ranging from .32 to .67 demonstrate that the items worked well together to produce a homogeneous scale. The alpha coefficient [45] demonstrated a high level of internal consistency reliability (α = .86, Mean = 32.6, SD = 8.3).

The second step in data analysis took an overview of the two items concerning religious practice and the three items concerning religiosity among Romanian Orthodox adolescents. These data are presented in Table 3.

The third step in data analysis explored the bivariate correlations between the two attitudinal measures (concerning socio-economic human rights, and euthanasia and abortion), the two personal factors (sex and age), the two measures of religious practice (worship attendance and personal prayer) and the three measures of religiosity (religious saliency, religious openness, and religious belief). These data are presented in Table 4. Three features of these bivariate correlations deserve comment. First, there are no significant age differences in respect of the two measures of attitude, the two measures of religious practice, and the three measures of religiosity. Second, there are significant sex differences. Females recorded higher scores affirming socio-economic rights (consistent with the findings of Francis, McKenna, and Sahin, [43]), higher scores permitting euthanasia and abortion (not consistent with Francis, McKenna, and Sahin, [42]), and higher scores on religious practice and religiosity (consistent with the general trends reported by Francis and Penny, [46]). Third, there is a complex pattern of correlations between religiosity and religious practice that require further clarification through regression modelling.

The fourth step in data analysis constructs a series of regression models with attitude as the dependent variable (SASHR in Table 5 and SAEA in Table 6), and with the independent variables being added incrementally in three steps. Model one begins by introducing the personal factors (sex and age). Model two adds the religious practices (worship attendance and personal prayer), Then, model three adds the religious measures (saliency, openness, and belief). For completeness Table 5 and Table 6 also incorporate the relevant correlations from Table 4 so that these can be read easily alongside the beta weights from the regression models.

Table 5 explores the effects of the independent variables on scores recorded on the Scale of Attitude toward Socio-economic Human Rights. Three features of these data deserve comment. First, the effect of sex remains constant across the three models. Females hold a more positive attitude toward socio-economic human rights, irrespective of the higher levels of religiosity and religious practice associated with being female. Second, in model 2 when religious practice is taken into account, there is a clear contrast between the effect of prayer and the effect of worship attendance. Higher levels of worship attendance (an indicator of extrinsic religiosity) are associated with lower endorsement of socio-economic human rights. Higher levels of personal prayer (as an indicator of intrinsic religiosity) are associated with higher endorsement of socio-economic human rights. Third, in model 3 when religiosity is taken into account, additional variance is accounted for by religious belief and religious saliency, two further indices that may be considered germane for intrinsic religiosity. When religious belief and religious saliency are in the model, the direct positive effect of personal prayer is now routed through these other two indices of intrinsic religiosity. The negative effect of worship attendance, however, remains consistent. This finding confirms the relative independence of the two streams of influence that we have characterised as indicators of intrinsic religiosity and of extrinsic religiosity.

Table 6 explores the effects of the independent variables on scores recorded on the Scale of Attitude toward Euthanasia and Abortion. Three features of these data deserve comment. First, the effect of sex remains constant across the models. Females are more in favour of permitting euthanasia and abortion. This sex difference holds true in spite of the findings that women tend to be more religious and that higher religiosity is associated with lower acceptance of euthanasia and abortion. Second, in model 2, when religious practice is taken into account, the effects of worship attendance and of personal prayer both work in the same direction. Higher frequency of worship attendance is associated with lower acceptance of euthanasia and abortion. Higher frequency of personal prayer is associated with lower acceptance of euthanasia and abortion. Moreover, the effects of worship attendance and personal prayer are cumulative in the sense that lowest acceptance of euthanasia and abortion is found among churchgoers who also pray. In other words, extrinsic religiosity and intrinsic religiosity are working in the same direction. Third, in model 3, when religiosity is taken into account, additional variance is accounted for by religious belief. When religious belief is in the model, the direct negative effect of personal prayer is now routed through religious belief. The negative effect of worship attendance, however, remains constant. This finding confirms the association between personal prayer and religious belief (as two indicators of intrinsic religiosity) functioning independently of worship attendance (as an indicator of extrinsic religiosity).

## 4. Conclusions

The present study set out to examine the complex connections linking religion, social attitudes, and attitude toward human rights among Romanian Orthodox adolescents. Within the specific social context of contemporary Romania and within the theological context of contemporary Orthodox Christianity, two contrasting sets of hypotheses were shaped to propose the connections between the two contrasting religious orientations (intrinsic religiosity and extrinsic religiosity) and two contrasting attitudinal areas (attitude toward socio-economic human rights and attitude toward euthanasia and abortion). In respect of attitude toward socio-economic human rights, it was hypothesised that high levels of church attendance (extrinsic religiosity) would be associated with lower endorsement of socio-economic human rights, while high levels of personal prayer (intrinsic religiosity) would be associated with higher endorsement of socio-economic human rights. In respect of attitudes toward euthanasia and abortion, it was hypothesised that high levels of church attendance (extrinsic religiosity) would be associated with lower endorsement of euthanasia and abortion, while high levels of personal prayer (intrinsic religiosity) would also be associated with lower endorsement of euthanasia and abortion. Moreover, it was further hypothesised that these two effects would be cumulative, so that low levels of endorsement of euthanasia and abortion would be associated with high frequency of church attendance coupled with high frequency of personal prayer. These two contrasting sets of hypotheses were tested on data provided by 400 Romanian Orthodox adolescents between the ages of 15 and 18 years who participated within the International Empirical Research Program Religion and Human Rights 2.0.

The first core finding from these data concerned the relative strength of extrinsic religiosity (as reflected in frequency of church attendance) and intrinsic religiosity (as reflected in frequency of personal prayer) among Romanian Orthodox adolescents. While all the adolescents included in these analyses self-identified as Orthodox Christians, the level of participation in religious services was relatively low: just 23% reported attending services at least once a month, of whom 11% reported attending services at least once a week. The level of engagement with personal prayer was much higher: 65% reported praying at least once a week, of whom 42% reported praying at least once a day. The findings related to church attendance are consistent with current nationally-representative research on Romanian youth [47], whereas the figures on prayer are relatively higher in the present study (65% compared to 42% at national level).

In other words, the adolescents who identify with the majority Orthodox denomination and who attend well-established educational settings, differ from the general Romanian youth by deeper levels of prayer life, whereas in terms of church attendance, they are roughly at the same level. Using the intrinsic-extrinsic differentiation, we can perhaps conclude that church attendance is not necessarily seen as an indicator of deeper religiosity for Romanian adolescents, but rather mere conformity with common social practice and norms. One element that makes a difference based on this study is the intrinsic personal appropriation of faith reflected in a more engaged life of prayer. Attending church services might simply be interpreted, like for the adult population of Romania, a rather external, cultural matter with less relevance for daily life, social or bioethical issues. The intrinsic dimension of religiosity reflected in a more pious personal life of prayer seems to be the differentiating factor, as the other main finding of this study shows.

Thus, the second core finding from the current data supported the two contrasting sets of hypotheses, and in so doing offered novel insights into the ways in which the strong presence of the Orthodox Christian tradition is operating within the lives of Romanian adolescents. These novel insights emerge from the same distinction between intrinsic religiosity and extrinsic religiosity and from the distinction between socio-economic human rights and the field of euthanasia and abortion.

In terms of attitude toward socio-economic human rights, it is clear that the effects of extrinsic religiosity and intrinsic religiosity work in opposite directions. Romanian Orthodox adolescents who identify closely with the Church by frequent church attendance are less supportive of socio-economic human rights. This finding shows conformity with socially dominant perspectives at national level, being along the same lines with current data on Romanian adolescents. The study run at national level mentioned above finds, for example, that 62% of the respondents think that women have too many/enough rights, 70% think that ethnic minorities have too many/enough rights, and 57% think the same about LGBT minorities, while 75% do not trust the government [47].

On the other hand, our study found that Romanian Orthodox adolescents who identify closely with the Christian tradition by frequent personal prayer are more supportive of socio-economic human rights. This finding is also in line with earlier studies which found that personal belief in God—another intrinsic dimension, alongside prayer—was a strong predictor of higher support for socio-economic rights amongst Romanian adolescents [11]. Religious piety that takes faith as a goal in itself seems to lead to deeper respect for otherness and deeper care for the “neighbor”, as reflected in the responses to the various forms of socio-economic human rights assessed in this study. It is in fact a reflection of “loving thy neighbor”, one of the central tenets of the Orthodox Christian teachings and tradition.

In terms of attitudes toward euthanasia and abortion, it is clear that the effects of extrinsic religiosity and intrinsic religiosity work in the same direction. Romanian Orthodox adolescents who identify closely with the Church by frequent church attendance are less supportive of euthanasia and abortion. At the same time, Romanian Orthodox adolescents who identify closely with the Christian tradition by frequent personal prayer are also less supportive of euthanasia and abortion. As seen in this study, this is consistent both with the popular Orthodox teachings and the general social culture on bioethics, being in line with figures relevant at national level among Romanian adolescents. For instance, 36% consider abortion as “never justified” and the numbers go up to 49% in the case of those who attend church often/very often [47].

Moreover, the effect is cumulative with the least support given for euthanasia and abortion being among adolescents who both attend public services frequently and engage in personal prayer frequently. This finding further strengthens the validity of the intrinsic-extrinsic assessment of religiosity, expressing stronger levels of conformity when applied both to the pious religious teachings, and to the dominant established social attitudes.

There are three main limitations with the present study. The first limitation concerns the relatively small sample size, limited to 400 participants, that did not permit contrasts to be made among different social settings. The findings are sufficiently intriguing, however, to warrant further replication and extension of the present study.

The second limitation concerns restricting the study to just two outcome measures, focusing on one aspect of human rights (socio-economic rights) and one aspect of social attitudes (euthanasia and abortion). Further developments from the present study would benefit from including a wider range of outcome measures.

The third limitation concerns restricting the measurement of intrinsic religiosity and extrinsic religiosity to proxy behavioural measures (frequency of public church attendance and frequency of personal prayer). Further developments from the present study would benefit from including richer measures of intrinsic religiosity and extrinsic religiosity, as operationalised, for example, by the New Indices of Religious Orientation [39].

## Figures and Tables

**Table 1:** Scale of Attitude toward Socio-economic Human Rights (SASHR): Scale properties.

	*r*	Yes (%)
*State’s obligation regarding the right to work*		
The government should provide a job for everybody who wants one	.72	74
The government should provide a decent standard of living for the unemployed	.44	55
*State’s obligation regarding the right to social security*		
The government should provide health care for the sick	.76	82
The government should provide a decent standard of living for the elderly	.68	76
*State’s obligation regarding living wages*		
Everyone should have the right to equal pay for equal work	.62	68
Everyone should have the right to a fair wage for their work	.73	78
*State’s obligation regarding rest and leisure*		
Everyone should have the right to a reasonable limitation on working hours	.75	80
Employment without paid holiday leave should be forbidden	.24	41
*State’s obligation regarding the rights of children*		
The state should protect children from forced labour	.71	79
The state should protect children’s right to play and recreation	.73	78
*State’s obligation regarding the protection of women from discrimination*		
The state should protect women’s rights to adequate job opportunities	.48	79
Women should have the right to equal pay for equal work	.58	82

Note: *r*, correlation between individual item and sum of other eleven items. %, sum of agree strongly and agree responses.

**Table 2:** Scale of Attitude toward Euthanasia and Abortion (SAEA): Scale properties.

	*r*	%
Euthanasia should be prohibited in all circumstances *	.32	41
Euthanasia should be permitted in the case of unbearable and irreversible suffering	.46	63
Euthanasia should be permitted in the case of unbearable and irreversible suffering if palliative care is exhausted	.55	56
Abortion should be prohibited in all circumstances because it ends human life *	.57	40
Abortion should be permitted in the case of rape	.67	55
Abortion should be permitted in the case of incest	.67	47
Abortion should be permitted when there is a strong chance of serious defects to the baby	.64	52
Abortion should be permitted when the woman’s own health is seriously endangered by the pregnancy	.62	60
Abortion should be permitted when the woman cannot afford more children economically	.62	29
Abortion should be permitted when the woman cannot afford more children psychologically	.63	31

Note: *r*, correlation between individual item and sum of other nine items. %, sum of agree strongly and agree responses. * These items were reverse coded to calculate *r* and the scale score.

**Table 3:** Religiosity measures and religious practice measures.

		%
How often do you think about religious issues?	Never	16
	Rarely	27
	Occasionally	32
	Often	19
	very often	6
How often do you reconsider certain aspects of your religious views?	Never	14
	Rarely	35
	occasionally	34
	often	12
	very often	5
I believe that God is the foundation of everything that exists	totally disagree	8
	disagree	11
	not sure	28
	agree	24
	fully agree	29
How often do you take part in religious services at a church or mosque or another place?	never	10
	hardly ever	17
	a few times a year	49
	1–3 times a month	12
	once a week	9
	more than once a week	2
How often do you pray?	never	7
	hardly ever	9
	a few times a year	11
	1–3 times a month	9
	once a week	9
	more than once a week	14
	once a day	32
	several times a day	10

**Table 4:** Correlation matrix.

	SAEA	SASHR	BEL	WOR	PRA	OPE	SAL	Sex
Age	.02	−.03	−.00	−.05	−.07	.07	.01	.04
Sex	.14 **	.23 ***	.18 ***	.10 *	.16 ***	.11 *	.11 *	
Saliency (SAL)	−.16 **	.18 ***	.35 ***	.32 ***	.39 ***	.66 ***		
Openness (OPE)	−.04	.13 *	.22 ***	.14 **	.28 ***			
Prayer (PRA)	−.25 ***	.14 **	.65 ***	.53 ***				
Worship (WOR)	−.34 ***	−.04	.43 ***					
Belief (BEL)	−.30 ***	.18 ***						
SASHR	.17 **							

Note: * *p* < .05; ** *p* < .01; *** *p* < .001. SASHR, Scale of Attitude toward Socio-economic Human Rights. SAEA, Scale of Attitude toward Euthanasia and Abortion.

**Table 5:** Regression models on Scale of Attitude toward Socio-economic Human Rights (SASHR).

	*r*	Model 1	Model 2	Model 3
*Personal factors*				
Sex	.23 ***	.23 ***	.21 ***	.20 ***
Age	−.03	−.02	−.01	−.02
*Religious practices*				
Worship attendance	−.04		−.17 **	−.21 ***
Personal prayer	.14 **		.19 ***	.08
*Religiosity*				
Religious belief	.18 ***			.13 *
Religious saliency	.18 ***			.17 *
Religious openness	.13 *			−.03
Total *r*^2^		.05	.08	.12
∆		.05 ***	.03 ***	.03 **

Note: * *p* < .05; ** *p* < .01; *** *p* < .001.

**Table 6:** Regression models on Scale of Attitude toward Euthanasia and Abortion (SAEA).

	*r*	Model 1	Model 2	Model 3
*Personal factors*				
Sex	.14 **	.14 **	.19 ***	.21 ***
Age	.02	.02	.00	.01
*Religious practices*				
Worship attendance	−.34 ***		−.29 ***	−.25 ***
Personal prayer	−.25 ***		−.13 *	.00
*Religiosity*				
Religious belief	−.30 ***			−.21 ***
Religious saliency	−.16 **			−.06
Religious openness	−.04			.05
Total *r*^2^		.02	.16	.19
∆		.02 *	.14 ***	.03 **

Note: * *p* < .05; ** *p* < .01; *** *p* < .001.

## Data Availability

The data are available upon request from the corresponding author.
